# Discovering Cancer-Related miRNAs from miRNA-Target Interactions by Support Vector Machines

**DOI:** 10.1016/j.omtn.2020.01.019

**Published:** 2020-01-25

**Authors:** Cong Pian, Shanjun Mao, Guangle Zhang, Jin Du, Fei Li, Suet Yi Leung, Xiaodan Fan

**Affiliations:** 1 Department of Statistics, The Chinese University of Hong Kong, Hong Kong SAR, China; 2 Department of Mathematics, College of Science, Nanjing Agricultural University, Nanjing, China; 3 Ministry of Agriculture Key Lab of Agricultural Entomology, Institute of Insect Sciences, College of Agriculture and Biotechnology, Zhejiang University, Hangzhou, China; 4 Department of Pathology, The University of Hong Kong, Queen Mary Hospital, Pokfulam, Hong Kong SAR, China; 5 Binjiang College, Nanjing University of Information Science and Technology, Jiangsu 214105, China

**Keywords:** cancer-related miRNAs, support vector machine, dark matters, miRNA-target interactions, expression data

## Abstract

MicroRNAs (miRNAs) have been shown to be closely related to cancer progression. Traditional methods for discovering cancer-related miRNAs mostly require significant marginal differential expression, but some cancer-related miRNAs may be non-differentially or only weakly differentially expressed. Such miRNAs are called dark matters miRNAs (DM-miRNAs) and are targeted through the Pearson correlation change on miRNA-target interactions (MTIs), but the efficiency of their method heavily relies on restrictive assumptions. In this paper, a novel method was developed to discover DM-miRNAs using support vector machine (SVM) based on not only the miRNA expression data but also the expression of its regulating target. The application of the new method in breast and kidney cancer datasets found, respectively, 9 and 24 potential DM-miRNAs that cannot be detected by previous methods. Eight and 15 of the newly discovered miRNAs have been found to be associated with breast and kidney cancers, respectively, in existing literature. These results indicate that our new method is more effective in discovering cancer-related miRNAs.

## Introduction

MicroRNAs (miRNAs) represent a type of small non-coding RNA molecule with about 22 nucleotides found in plants, animals, and viruses that function in post-transcriptional regulation of gene expression and RNA silencing by binding to the 3′ untranslated regions of mRNA.^1^, ^2^, ^3^, ^4^ miRNAs are abundant in many mammalian cells^5^^,^^6^ and appear to target about 60% of the genes of mammals.^7^^,^^8^ Many miRNAs are evolutionarily conserved, which indicates that they have significant biological functions.^9^ Research suggests that miRNAs can act as regulators of diverse cellular processes, such as cell differentiation, apoptosis, virus defense, embryonic development, and proliferation.^10^^,^^11^ Furthermore, miRNAs have been implicated in many diseases, such as various types of cancers,^12^, ^13^, ^14^ heart conditions,^15^ and neurological diseases.^16^ Up to now, miRNAs have been studied as promising candidates for diagnostic and prognostic biomarkers, as well as predictors of drug responses. For example, miR-1246 is a potential diagnostic and prognostic biomarker in esophageal squamous cell carcinoma (ESCC), and may act as a cell adhesion-related miRNA released from ESCC that affects distant organs.^17^ Research shows that single-nucleotide polymorphisms (SNPs) in miRNAs and their target sites can impact miRNA biology and affect cancer risk, as well as treatment response.^18^ It is likely that these SNPs can act as diagnostic and prognostic markers. Thus, discovering pivotal cancer-related miRNAs is an active area of research.

**Figure 1:**
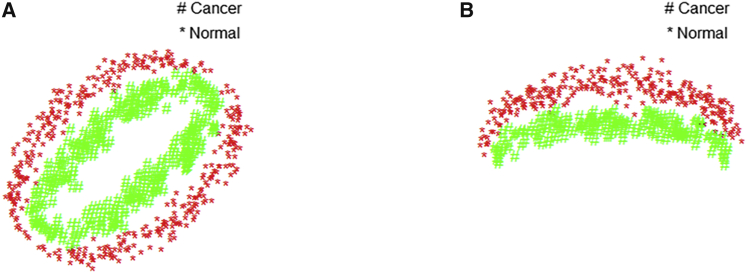
Two Situations that ΔPCC Has Difficulty Handling Points of two colors represent samples from the normal and cancer groups. (A) Consistent correction through embedding. (B) Nonlinear association.

The differential expression analysis (DE), which performs two groups comparison for individual miRNA followed by certain multiple comparison correction, may be the most common method of discovering cancer-related miRNAs. For example, in Zhou et al.,^19^ differentially expressed miRNAs and mRNAs were separately selected as biomarkers using the limma package; in Liao et al.,^20^ 5 miRNAs of 320 differentially expressed mRNAs were used for prognostic signature construction; in Le et al.,^21^ a causality discovery-based method was used to uncover the causal regulatory relationship between miRNAs and mRNAs. However, some non-differentially or weak differentially expressed miRNAs may play important regulatory roles in cancer. Pian et al.^22^ named this type of miRNA “dark matters” miRNA (DM-miRNA) and developed a method to discover DM-miRNA based on the change of Pearson correlation coefficient (ΔPCC). However, ΔPCC may fail in some situations. For example, if the correlations between a miRNA and its target in cancer and normal samples are consistent as in Figure 1A, ΔPCC will be too small to discover this MTI. Also, ΔPCC is based on Pearson correlation, which cannot detect nonlinear associations, such as in Figure 1B.

Here, we introduce a machine learning method to discover cancer-related miRNAs. More specifically, support vector machines (SVMs) are used to construct nonlinear class separation boundaries in the two-dimensional space of a miRNA and its experimentally validated target. By focusing on experimentally validated miRNA-target interactions (MTIs), we can avoid many false positives as compared with the DE method on marginal expression. With the ability of SVMs to induce complex decision boundaries, we can accommodate nonlinear or even embedded class relationships as in Figure 1. The classification accuracy (ACC, see definition in Materials and Methods) is used to screen signals and compare different approaches.

## Results

### Results for Breast Cancer

#### miRNAs with High Classification Accuracy (S_1_)

**Figure 2:**
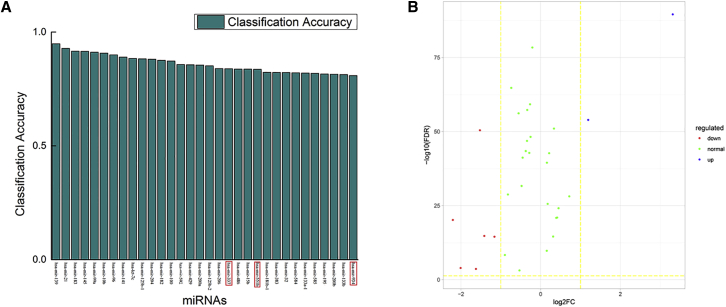
The 32 miRNAs with ACC >0.8 in Breast Cancer (A) The relationship between the 32 miRNAs and breast cancer. The miRNAs in the red rectangular boxes are so far not experimentally confirmed to be associated with BRCA. (B) The volcano map of the above 32 miRNAs. Most of these miRNAs are not differentially expressed.

**Figure 3:**
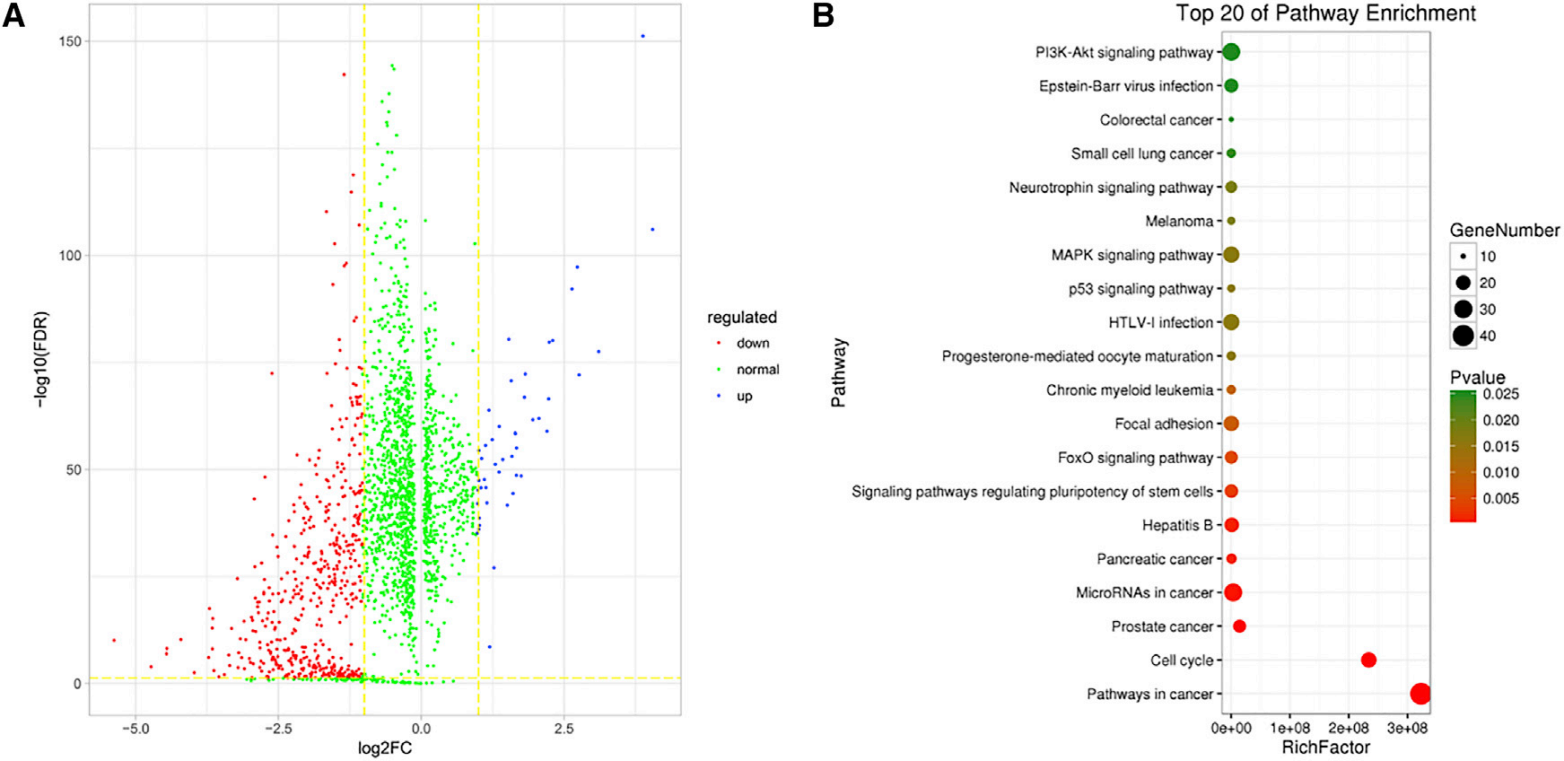
The 2,028 mRNAs Whose ACCs Are Greater Than 0.8 for Breast Cancer (A) The volcano map of 2,028 mRNAs. Red and blue represent downregulation and upregulation, respectively. (B) The enrichment analyses result of the above 2,028 mRNA genes based on KEGG pathways for BRCA.

We use the breast cancer expression data of each miRNA as the input feature to train an SVM classifier. Figure 3A shows the miRNAs whose ACC is greater than 0.8. The miRNAs in the red rectangular boxes are not experimentally confirmed to be associated with breast invasive carcinoma (BRCA). The remaining miRNAs have been shown to be associated with breast cancer based on the database HMDD 2.0 and literature mining. The PubMed numbers of these miRNAs are shown in Table 1. Figure 2B is the volcano map of miRNAs in Figure 2A. We find that most of these miRNAs are not differentially expressed. The results indicate that the SVM based on miRNA expression data alone can discover partial BRCA-related miRNAs.

**Table 1:** The Literature Reports of the Associations between the miRNAs with ACC >0.8 on miRNA Expression and Breast Cancer

miRNA	PubMed No.
miR-139	21953071
miR-21	17531469
miR-183	23060431
miR-145	21723890
miR-99a	27212167
miR-10b	22573479
miR-96	19574223
miR-141	18376396
let-7c	22388088
miR-125b-1	19738052
miR-204	18922924
miR-182	19574223
miR-100	22926517
miR-592	29039599
miR-429	18376396
miR-200a	20514023
miR-125b-2	20460378
miR-206	17312270
miR-337	unknown
miR-486	19946373
miR-15b	25783158
miR-551b	unknown
miR-181b-1	23759567
miR-383	16754881
miR-32	26276160
miR-584	23479725
miR-133a-1	22292984
miR-585	22328513
miR-195	30076862
miR-200b	20514023
miR-133b	19946373
miR-934	unknown

#### miRNAs with High Classification Accuracy (S_2_)

We also use the breast cancer expression data of each mRNA as the input feature to train an SVM classifier. Figure 3A describes the DE results of 2,028 mRNAs whose ACCs are greater than 0.8. In addition, the enrichment analyses results are shown in Figure 3B. DAVID^23^^,^^24^ is employed for enrichment analyses for the above 2,028 mRNAs based on Kyoto Encyclopedia of Genes and Genomes (KEGG) pathways. Some cancer mechanism-related pathways (such as pathways in cancer and the p53 signaling pathway, prostate cancer, miRNAs in cancer, pancreatic cancer, chronic myeloid leukemia, melanoma, the p53 signaling pathway, small cell lung cancer, colorectal cancer) are significantly enriched. These results indicate that the discovered mRNAs are very important in cancers.

#### MTIs with High Classification Accuracy (S_3_)

For each of the 155,044 experimentally verified human MTIs from the miRTarBase database, we use the mRNA and miRNA breast cancer expression data of the miRNA-mRNA interaction as the two features of SVM. The MTIs with high ACC >0.8 are selected as candidate MTIs for discovering cancer-related miRNAs.

#### Discovery of DM-miRNAs in Breast Cancer

**Figure 4:**
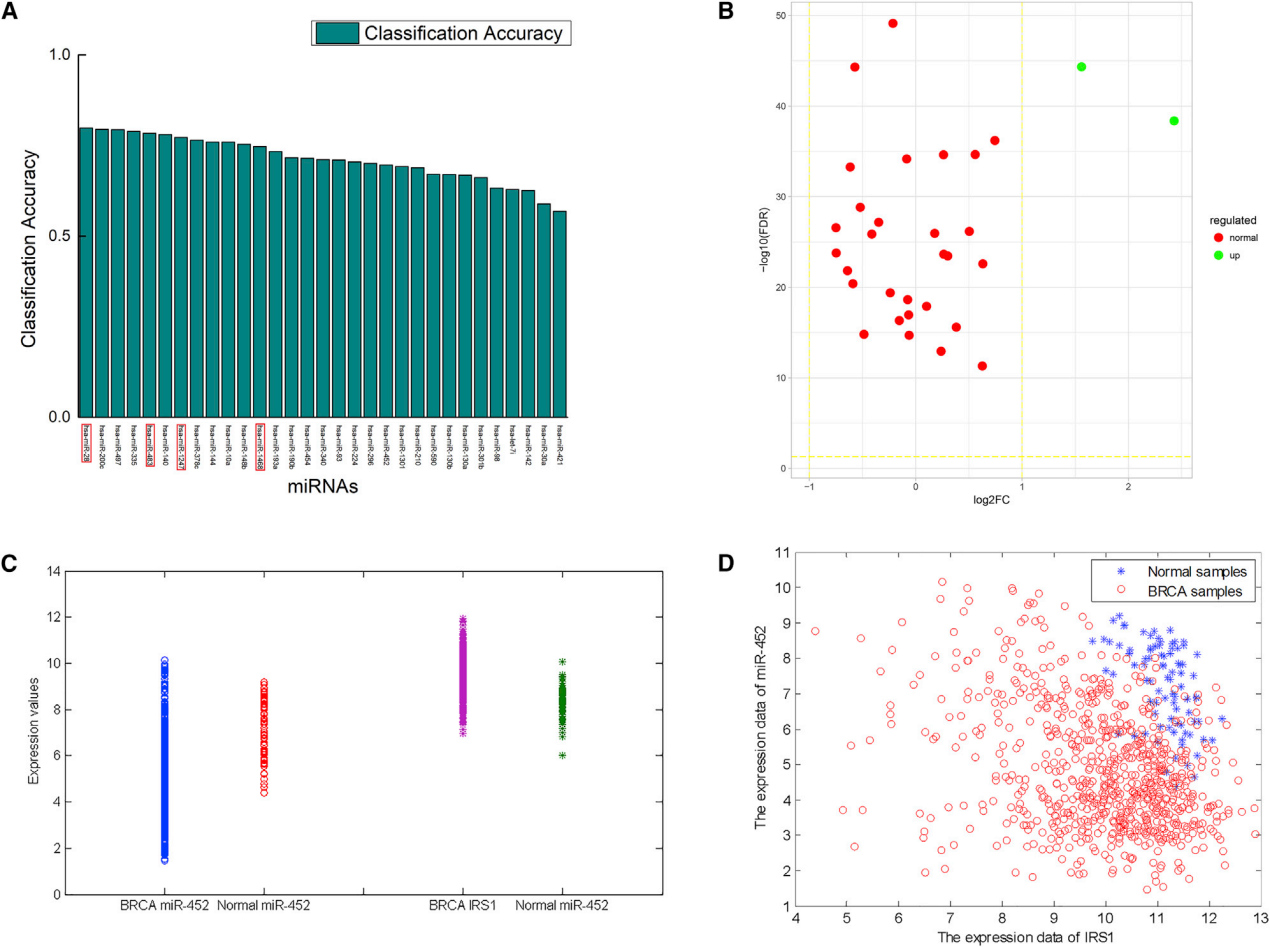
The 31 miRNAs in 136 MTIs with [ACC(miRNA-mRNA) > 0.9, ACC(miRNA) < 0.8, ACC(mRNA) < 0.8] for Breast Cancer (A) The relationship between these miRNAs and cancer. The miRNAs in the red rectangular boxes are not experimentally confirmed to be associated with BRCA. (B) The volcano map of the above 31 miRNAs. Only 2 of 31miRNAs are differentially expressed. (C) The one-dimensional scatterplot of single miR-452 and IRS1 expression values in normal and cancer samples. The left two lines represent the expression value of miR-452 in BRCA and normal tissues, and the right two lines represent the expression value of IRS1 in BRCA and normal tissues. (D) The two-dimensional scatterplot of miRNA-mRNA interaction. The abscissa and ordinate represent the expression values of IRS1 and miR-452.

To demonstrate why our new method can catch better discriminant information, we analyze the MTIs with ACC >0.9 in the miRNA-mRNA joint space, whereas the corresponding marginal ACC of both the miRNA and the mRNA are <0.8. There are 136 MTIs satisfying the above conditions (Table S2). Thus, although the ACCs based on the marginal miRNA feature and the marginal mRNA feature are both nonideal, the performance of classification of the corresponding MTI, i.e., the joint feature, is significant. Figure 4A shows the 31 miRNAs in 136 MTIs. The miRNAs in the red rectangular boxes are so far not experimentally confirmed to be associated with BRCA. The PubMed numbers of these miRNAs are shown in Table 2. We see that most of these 31 miRNAs are related to BRCA and non-differentially expressed in Figure 4B. There are two differentially expressed miRNAs. Figure 4C represents the expression of miR-452 and IRS1 in normal and cancer samples. We find that it is hard to distinguish the normal and cancer samples based only on the feature of single miRNA or only on the mRNA expression profile data. More specifically, the classification accuracy of using miR-452 or IRS1 alone is 69.61% or 62.55%, respectively. Figure 4D is the scatterplot of miR-452 and IRS1. Compared with the classification performance of either marginal feature miR-452 or IRS1, the detection using the two-dimensional features of miR-452 and IRS1 is much more effective.

**Table 2:** The Literature Reports of the Associations between DM-miRNAs and Breast Cancer

miRNA	PubMed No.
miR-28	unknown
miR-200c	21224848
miR-497	27456360
miR-335	28795314
miR-483	30186493
miR-140	23752191
miR-1247	30249392
miR-378c	26749280
miR-144	29561704
miR-10a	21955614
miR-148b	23233531
miR-1468	unknown
miR-193a	22333974
miR-190b	26141719
miR-454	27588500
miR-340	21692045
miR-93	21955614
miR-224	22809510
miR-296	19754881
miR-452	22353773
miR-1301	29790898
miR-210	22952344
miR-590	29534690
miR-130b	28163094
miR-130a	29384218
miR-301b	21393507
miR-98	28232182
let-7i	22388088
miR-142	26657485
miR-30a	22231442
miR-421	28463794

The underlined miRNAs are experimentally confirmed.

If we relax the thresholds in the previous paragraph by analyzing the MTIs with ACC >0.8 in the joint feature and ACC <0.7 in both marginal features, the results are shown in Table 3. The underlined miRNAs are experimentally confirmed to be associated with BRCA. The second and third columns are the fold change (FC) and PubMed numbers of literature reports of these miRNAs, respectively. Most of these miRNAs are not differentially expressed.

**Table 3:** The FC and Literature Reports of miRNA [ACC(miRNA-mRNA) > 0.8, ACC(miRNA) < 0.7, ACC(miRNA) < 0.7)] for Breast Cancer

miRNA	FC	PubMed No.
miR-30a	0.065	22476851
miR-331	0.343	30063890
miR-23b	0.015	22231442
miR-17	0.091	18695042
miR-92a-2	0.036	22563438
miR-449a	3.004	27983918
miR-134	0.095	28454346
let-7b	0.035	22403704
miR-127	0.080	21409395
miR-3127	0.507	unknown
miR-20a	0.018	22350790
miR-30c-2	0.070	23340433
miR-421	0.627	28463794
miR-125a	0.052	23420759
miR-186	0.048	unknown
miR-877	1.131	unknown
miR-222	0.062	21553120
miR-330	0.234	29630118

The underlined miRNAs are experimentally confirmed.

In summary, compared with the single miRNA or mRNA, paired MTIs contain more biological information. Therefore, the SVM classifier based on the paired miRNA-mRNA features can effectively discover more DM-miRNAs.

**Figure 5:**
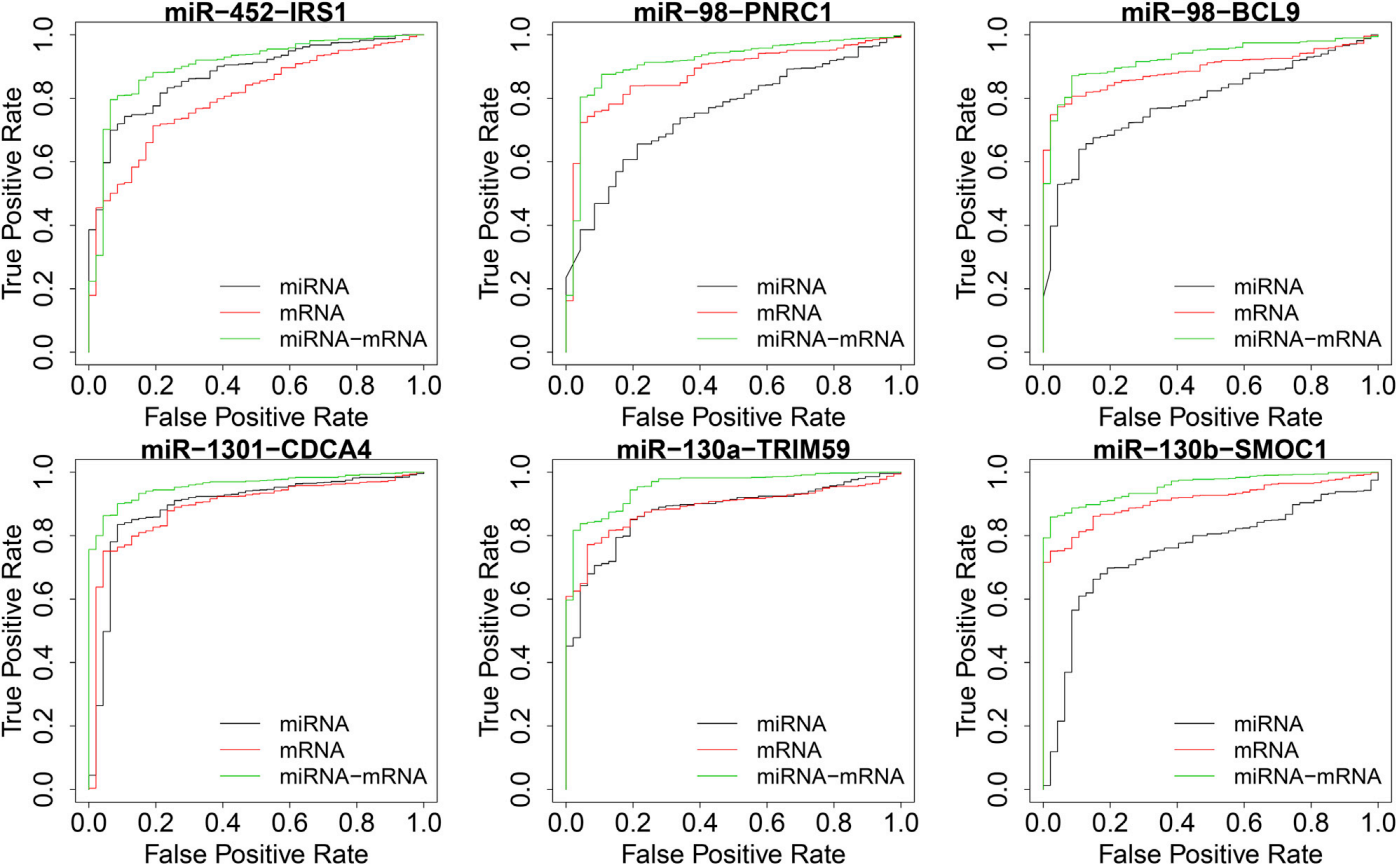
The ROC Curves of Six MTIs with ACC >0.9 for Breast Cancer The classification results of miR-452-IRS1, miR-98-PNRC1, miR-98-BCL9, miR-1301-CDCA4, miR-130a-TRIM59, and miR-130b-SMOC1. The black and red lines represent the ROC curve based on the single miRNA and mRNA, respectively. The green line represents the ROC curve based on the paired miRNA-mRNA interaction.

We draw receiver operating characteristic (ROC) curves by randomly selecting six MTIs with ACC >0.9 [ACC(miRNA) < 0.8, ACC(mRNA) < 0.8]. Figure 5 shows the classification performance based on the single mRNA, miRNA, and paired MTIs for BRCA. The results indicate that the information of MTIs is more effective. The classification ability of MTIs is significantly better than that of mRNAs and miRNAs. Therefore, MTIs can be effective biomarkers that contain more biological information.

#### Comparison with DE of miRNAs

In order to show that SVM can effectively screen potential cancer-related miRNAs, we compared the results of SVM and DE. Table 4 records the top 20 |log2(FC)| miRNAs in breast cancer based on the DE. The results in Table 5 indicate that only 4 of the top 20 miRNAs were confirmed to be associated with breast cancer. The underlined miRNAs are experimentally confirmed to be associated with BRCA. However, Table 2 shows that 19 of the top 20 ACC miRNAs were confirmed to be associated with breast cancer, which indicates that using SVM to select cancer-related miRNAs is more effective.

**Table 4:** The Top 20 |log2(FC)| miRNAs in Breast Cancer

miRNA	|log2(FC)|	PubMed No.^a^
miR-802	5.412	26080894
miR-449c	4.186	unknown
miR-3927	4.764	unknown
miR-3139	4.608	unknown
miR-124-2	4.458	unknown
miR-492	4.324	25407488
miR-573	4.253	25333258
miR-1908	4.253	unknown
miR-549	4.084	unknown
miR-3156-2	4.034	unknown
miR-3156-1	4.034	unknown
miR-507	4.031	27167339
miR-3180	4.017	unknown
miR-3612	3.982	unknown
miR-3925	3.829	unknown
miR-1302-3	3.677	unknown
miR-449b	3.580	unknown
miR-3156-3	3.569	unknown
miR-3148	3.568	unknown
miR-592	3.349	unknown

The underlined miRNAs are experimentally confirmed to be associated with BRCA.

a The third column represents the PubMed number of literature reports of these miRNAs.

**Table 5:** The Literature Reports of the Associations between DM-miRNAs and Kidney Cancer

miRNA	PubMed No.
let-7b	28694731
let-7g	25951903
let-7i	28694731
mir-100	28765937
mir-154	30138594
mir-15b	unknown
mir-183	26091793
mir-186	28550686
mir-20b	26708577
mir-214	27226530
mir-216b	30231239
mir-23b	20562915
mir-26a-1	28881158
mir-30b	28536082
mir-320a	27760486
mir-335	29070041
mir-340	unknown
mir-369	unknown
mir-377	25776481
mir-483	unknown
mir-493	unknown
mir-513c	unknown
mir-625	unknown
mir-675	unknown

The underlined miRNAs are experimentally confirmed to be associated with kidney cancer.

#### Results for Kidney Cancer

For comparison with the previous method ΔPCC, we show the results for kidney cancer. As before, we analyze MTIs with ACC >0.9 and whose single miRNA and mRNA have ACC <0.8. A total of 76 such MTIs are selected (Table S3). Table 5 describes the mRNAs in these 76 MTIs. The underlined miRNAs are experimentally confirmed to be associated with kidney cancer. The PubMed numbers of these miRNAs are shown in the second and fourth columns of Table 5.

We also compare the results of SVM and DE in kidney renal clear cell carcinoma (KIRC). Table 6 records the top 20 |log2(FC)| miRNAs in kidney cancer based on DE. Table 7 records the top 20 ACC miRNAs in kidney cancer based on SVM classifier. The underlined miRNAs are experimentally confirmed to be associated with KIRC. Results in Table 6 indicate that only 3 of the top 20 miRNAs were confirmed to be associated with kidney cancer. However, Table 7 shows that 16 of the top 20 ACC miRNAs were confirmed to be associated with kidney cancer. These results also indicate that using SVM to select cancer-related miRNAs is more effective.

**Table 6:** The Top 20 |log2(FC)| miRNAs in Kidney Cancer

miRNA	|log_2_(FC)|	PubMed No.^a^
miR-1293	5.143	28338236
miR-122	5.007	23056576
miR-875	4.582	unknown
miR-3166	4.523	unknown
miR-3202-2	4.431	unknown
miR-1285-1	4.108	22294552
miR-1231	3.869	unknown
miR-1250	3.832	unknown
miR-520b	3.788	unknown
miR-518c	3.777	unknown
miR-3654	3.775	unknown
miR-219-2	3.704	unknown
miR-2115	3.602	unknown
miR-3617	3.484	unknown
miR-555	3.434	unknown
miR-548d-2	3.413	unknown
miR-3662	3.302	unknown
miR-1910	3.289	unknown
miR-597	3.278	unknown
miR-3941	3.199	unknown

The underlined miRNAs are experimentally confirmed to be associated with KIRC.

a The third column represents the PubMed number of literature reports of these miRNAs.

**Table 7:** The Top 20 ACC miRNAs in Kidney Cancer

miRNA	ACC (%)	PubMed No.^a^
miR-200c	98.75	29394133
miR-141	98.51	24647573
miR-206	95.53	29410711
miR-122	94.28	29410711
miR-129-1	94.10	24802708
miR-129-2	93.75	28251969
miR-629	93.21	25381221
miR-584	92.86	21119662
miR-891a	92.68	unknown
miR-106b	91.96	28423523
miR-210	91.96	29445446
miR-181b-1	91.43	unknown
miR-15a	90.89	28849086
miR-934	90.54	unknown
miR-21	90.53	29131259
miR-429	90.35	27698878
miR-151	90.00	unknown
miR-181a-1	89.82	29066014
miR-155	89.64	29228417
miR-25	89.64	29079415

The underlined miRNAs are experimentally confirmed to be associated with KIRC.

a The third column represents the PubMed number of literature reports of these miRNAs.

#### Identification of Cancer Types via miRNA-mRNA Association

**Figure 6:**
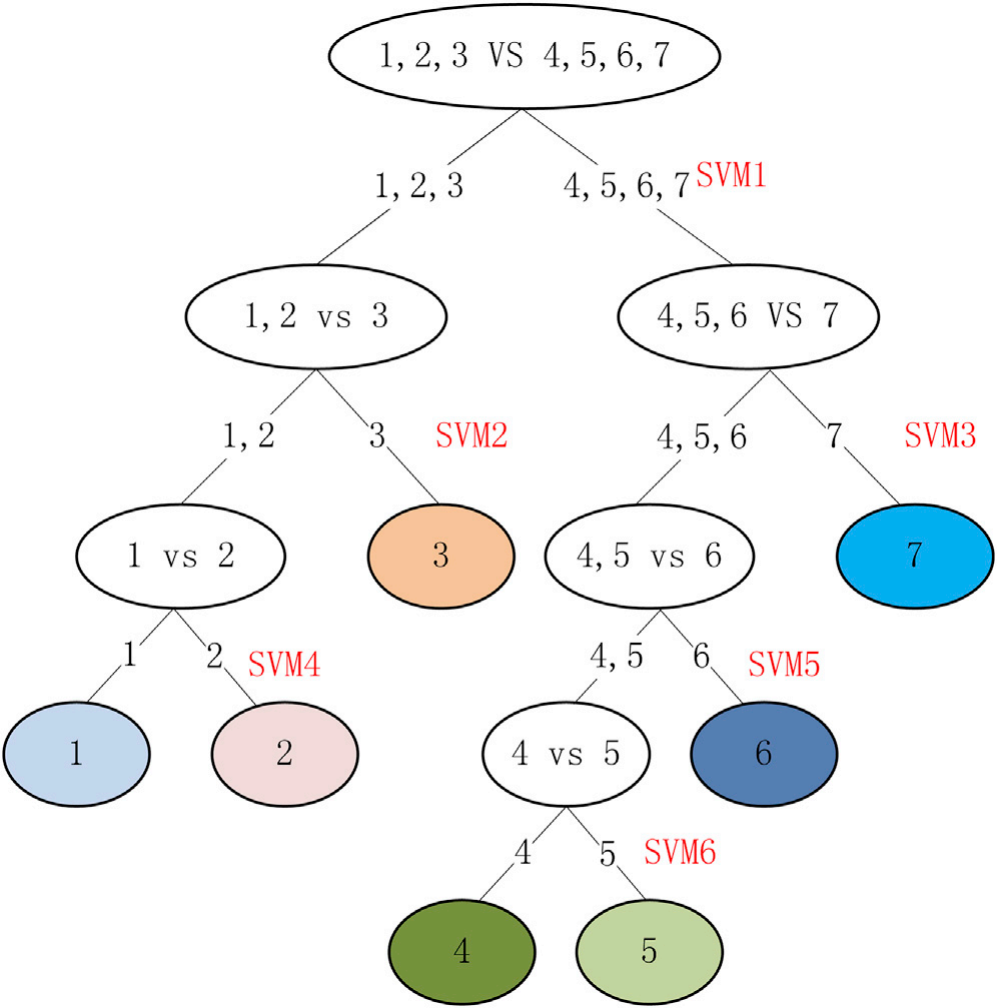
The Flow Chart for Constructing the Multiclass Classifier The numbers 1–6 represent LUSC, LUAD, BRCA, THCA, PRAD, and KIRC, respectively. The number 7 represents the normal tissue samples. The process contains six SVM classifiers. For sample S1, where the type of cancer is not known, if S1 is classified as “1,2,3” using SVM1, then we use SVM2 to judge its type. If S1 is classified as “3,” the final prediction type is BRCA, otherwise S1 needs to be further predicted through SVM4.

To verify whether miRNA-mRNA associations can effectively classify cancer types, we designed a multiclass classifier with multiple SVM sub-classifiers to identify the six cancers and the normal tissues. The miRNA-mRNA pairs with joint ACC >0.8 but marginal ACC <0.7 were selected as the features of the classifiers. The detailed flow chart is in Figure 6. The index “1–6” represents the six kinds of cancer (lung squamous cell carcinoma [LUSC], lung adenocarcinoma [LUAD], BRCA, thyroid carcinoma [THCA], prostate adenocarcinoma [PRAD], KIRC), respectively. The index “7” represents the integration of paired normal tissue samples. We divided these seven classes into two subclasses. Further subclasses are further divided into two subclasses, which are so circulated until a single class is obtained. Finally, we evaluated the performance of the classifier using 10-fold cross-validation. The accuracies of the seven classes are shown in Table 8. The diagonal elements are the percentages of real LUSC, LUAD, BRCA, THCA, PRAD, KIRC, and normal samples identified correctly. The remaining elements are the percentage of a class of samples judged to be the six types of samples. The results indicate that the miRNA-mRNA associations can be used to precisely identify cancer types.

**Table 8:** The Performance of the Multiclass Classifier by Using 10-Fold Cross-Validation

	LUSC	LUAD	BRCA	THCA	PRAD	KIRC	Normal
LUSC	97.28	0.81	0.29	0.16	0.28	0.54	0.64
LUAD	1.83	96.22	0.52	0.35	0.41	0.36	0.31
BRCA	0.12	0.29	97.16	0.84	042	0.58	0.59
THCA	0.34	0.47	0.46	97.38	0.73	0.39	0.23
PRAD	0.26	0.38	0.41	0.46	97.14	0.68	0.67
KIRC	0.52	0.32	0.37	0.43	0.46	97.42	0.48
Normal	0.24	0.33	0.34	0.28	0.52	0.15	98.14

#### Comparison with Other Methods

Pian et al.^22^ provided a method called ΔPCC to discover potential DM-miRNAs by building the basic miRNA-mRNA network (BMMN) and miRNA-long noncoding RNA (lncRNA) network (BMLN). For breast cancer, 124 miRNAs with high activity scores were obtained by BMMN. In this paper, we obtained 49 miRNAs by integrating Tables 2 and 3. Through comparing these 124 and 49 miRNAs, we found that 9 of 49 miRNAs (hsa-miR-331, hsa-miR-142, hsa-miR-3127, hsa-miR-222, hsa-miR-378c, hsa-miR-92a-2, hsa-miR-421, hsa-miR-125a, and hsa-miR-590) did not appear in the 124 miRNAs. Tables 2 and 3 show that all nine of the above miRNAs except hsa-miR-3127 have been confirmed to be associated with breast cancer. For kidney cancer, 70 miRNAs with high activity score were obtained by BMMN. Only one (miR-let-7b) of the 24 miRNAs in Table 5 appears in the above 70 miRNAs. Fifteen of the remaining 23 miRNAs have been confirmed to be associated with kidney cancer. The above results indicate that our new method can find cancer-related miRNAs that cannot be discovered by ΔPCC.

## Discussion

Cancers have a high incidence of occurrence globally. Their high mortality rates highlight the urgent need for new treatment methods. miRNAs are important post-transcriptional gene expression regulators. In cancer, the miRNAs aberrantly expressed have significant roles in progression and tumorigenesis. Currently, miRNAs are being studied as biomarkers for diagnosis and prognosis, and as therapeutic tools in cancer. However, some important miRNAs are easily overlooked, when the correlations between these miRNAs and their target genes in cancer and normal samples are consistent. In order to discover these miRNAs, we use a novel method to discover them by building SVM classifiers based on potential joint MTIs. Our results indicate that the new method can detect additional cancer-related miRNAs that cannot be detected by previous methods. Our new method should be considered complementary to previous methods. We also find that the edge biomarkers contain more biological information than the node biomarkers. Compared with the signal miRNA or mRNA biomarkers, edge biomarkers (paired miRNA-mRNA interaction) can more effectively distinguish tumor samples and normal samples. Furthermore, by constructing a classifier with multiple random forest sub-classifiers based on the edge biomarkers, the six cancers can be identified accurately. This will provide a new way to further study the classification of tumor sub-types. In conclusion, our method can help effectively discover new cancer-related miRNAs. These results will contribute to developing novel therapeutic candidates in cancers.

Our method also has some limitations. For example, our method is based on the known MTIs from miRTarBase;^25^ thus, it cannot detect newly gained MTIs that have not been recorded in miRTarBase. To remedy this potential loss, a systematic scan of all miRNA-mRNA pairs may be needed, which will be very computationally costly.

## Materials and Methods

### Datasets

We studied different types of cancer, including BRCA, KIRC, LUAD, LUSC, THCA, and prostate adenocarcinoma (PRAD). The expression profiles of these six cancers were downloaded from the database of The Cancer Genome Atlas (TCGA) (https://www.cancer.gov/about-nci/organization/ccg/research/structural-genomics/tcga), which includes 1,071 miRNAs and 20,530 mRNAs. The number of cancer samples is shown in Table 9. The 155,044 experimentally validated MTIs (Table S1) and miRNA-disease associations were obtained from the databases miRTarBase and HMDD v.2.0, respectively.^25^^,^^26^

**Table 9:** The Type and Sample Number of Six Different Types of Cancer

Cancer Abbreviation	Full Name of Cancer	No. of Cancer Tissue Samples	No. of Paired Normal Tissue Samples
BRCA	breast invasive carcinoma	755	86
KIRC	kidney renal clear cell carcinoma	255	71
THCA	thyroid carcinoma	511	59
LUAD	lung adenocarcinoma	445	19
LUSC	lung squamous cell carcinoma	342	38
PRAD	prostate adenocarcinoma	494	52

### Flow Chart of the Method

**Figure 7:**
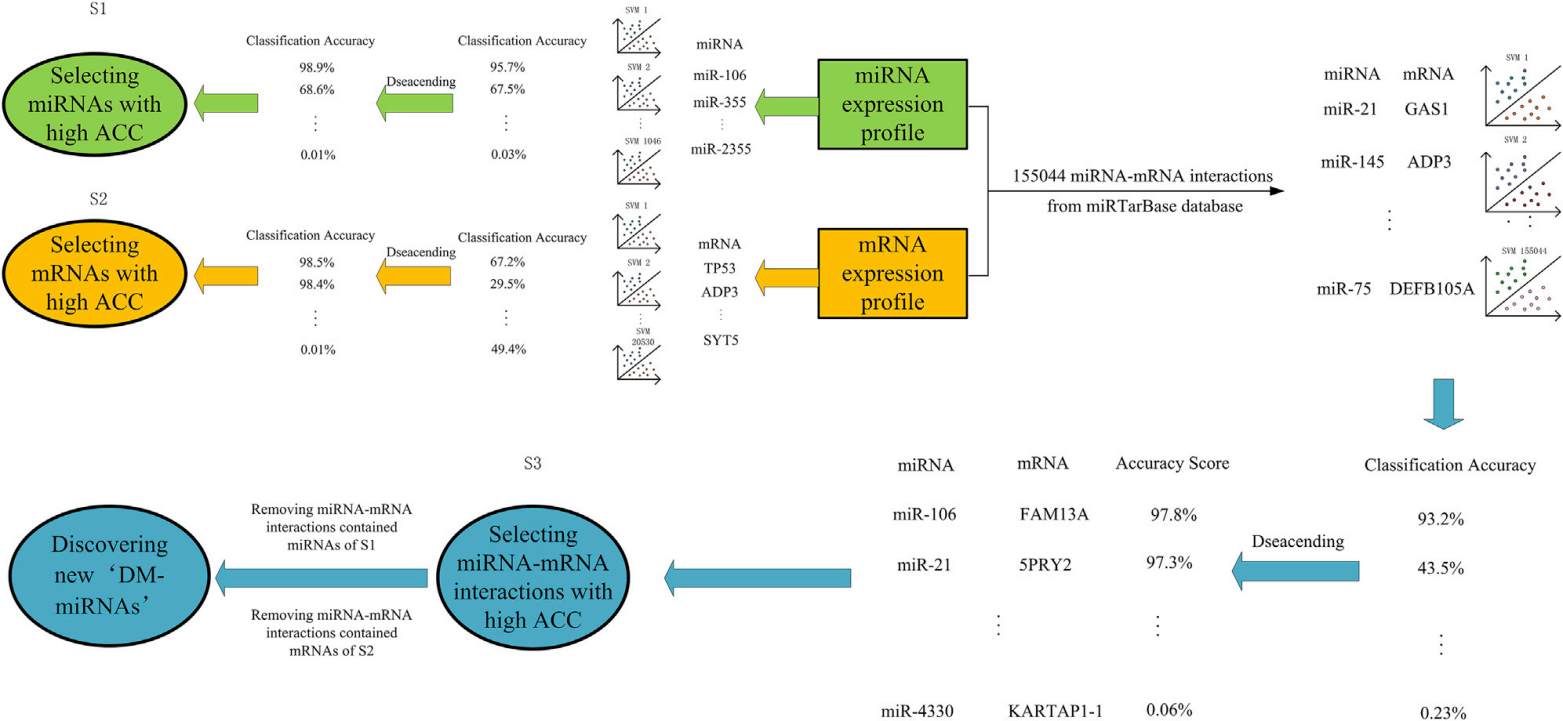
The Flow Chart of Our Method The green modules represent the SVM classification results based on the miRNA expression feature. The miRNAs with high ACC are selected as set S1. The orange modules represent the SVM classification results based on the mRNA expression feature. The mRNAs with high ACC are selected as set S2. The blue modules represent the SVM classification results based on the paired MTIs feature. We select paired MTIs with high ACC as set S3. DM-miRNAs are inferred as the MTIs of S3 after removing those containing miRNAs of S1 or mRNAs of S2.

The workflow of DM-miRNA discovery is divided into four steps (Figure 7). First, an SVM classifier is constructed for each of the 1,071 miRNAs based on its expression data in cancer and normal tissues. Therefore, the classification accuracy (ACC) based on each miRNA expression feature is obtained. We select miRNAs with high ACC as set S1. In step 2, likewise, ACC based on each mRNA expression feature is calculated by building 20,530 SVM classifiers. The mRNAs with high ACC are selected as set S2. In step 3, ACCs based on 155,044 paired miRNA-mRNA expression features are also obtained by building 155,044 SVM classifiers. We select paired miRNA-mRNA interactions with high ACC as set S3. Finally, we obtain potential DM-miRNAs by removing the MTIs of S3, which contain miRNAs of S1 or mRNAs of S2.

### Parameters of the Model

The kernel, cost, and gamma of SVM were set to radial, 1, and 1, respectively. Because the positive (86 normal samples) and negative samples (755 BRCA samples) were unbalanced, we used the random sub-sampling method to balance the data. We sampled the training set and the testing set 20 times. Each time, 40 positive samples and 40 negative samples were randomly chosen to form a training set. The corresponding test set is randomly selected from the remaining positive and negative samples, which guarantees that there is no overlap between the training and testing sets. The SVM classification accuracy (ACC) of the 20 groups of balanced data was obtained. We use the mean value of the 20 ACCs as the final accuracy. The formula for ACC from any testing data is defined as follows:

$$ACC=\frac{T_{P}+T_{N}}{T_{P}+F_{N}+F_{P}+T_{N}}\times 100\%,$$

where TP (true positive) is the number of positive samples that are identified correctly, FN (false negative) is the number of positive samples that are identified incorrectly, TN (true negative) is the number of negative samples that are identified correctly, and FP (false positive) is the number of negative samples that are identified incorrectly.

## Author Contributions

C.P. and X.F. conceived and designed the study; C.P., S.M., and G.Z. analyzed the data; J.D., S.Y.L., and F.L. contributed ideas and comments; C.P. and X.F. wrote the paper; and all authors read and approved the final manuscript.

## Conflicts of Interest

The authors declare no competing interests.

## Supplemental Information

Table S1. The 155,044 Experimentally Validated MTIs

Table S2. Prediction Accuracy and Identified Signals Based on BRCA Data Sheet1 in Table S2 records the predictive accuracy of 155,044 MTIs, 1,046 miRNAs and 20,530 mRNA based on the expression data of BRCA. Sheet2 represents 136 MTIs with ACC>0.9 in the miRNA-mRNA join space while the corresponding marginal ACC of both the miRNA are the mRNA are less than 0.8 in BRCA.

Table S3. Prediction Accuracy and Identified Signals Based on KIRC Data Sheet1 in Table S3 records the predictive accuracy of 155,044 MTIs, 1,046 miRNAs and 20,530 mRNA based on the expression data of KIRC. Sheet2 represents 76 MTIs with ACC>0.9 in the miRNA-mRNA join space while the corresponding marginal ACC of both the miRNA are the mRNA are less than 0.8 in KIRC
